# Similar expression to FGF (Sef) inhibits fibroblast growth factor-induced tumourigenic behaviour in prostate cancer cells and is downregulated in aggressive clinical disease

**DOI:** 10.1038/sj.bjc.6605379

**Published:** 2009-11-03

**Authors:** S Darby, T Murphy, H Thomas, C N Robson, H Y Leung, M E Mathers, V J Gnanapragasam

**Affiliations:** 1 Northern Institute for Cancer Research, University of Newcastle, Newcastle upon Tyne NE2 4HH, UK; 2 Translational Prostate Cancer Group, Department of Oncology, Hutchison MRC Research Centre, University of Cambridge, Cambridge CB2 OXZ, UK; 3 Beatson Institute, Garscube Estate, Glasgow, G61, UK; 4 Department of Pathology, Western General Hospital, Crewe Road, Edinburgh EH4 2XU, UK

**Keywords:** prostate cancer, fibroblast growth factor, similar expression to FGF (Sef), downregulation, aggressive disease

## Abstract

**Background:**

The fibroblast growth factor (FGF) axis is an important mitogenic stimulus in prostate carcinogenesis. We have previously reported that transcript level of human similar expression to FGF (hSef), a key regulator of this pathway, is downregulated in clinical prostate cancer. In this study we further analysed the role of hSef in prostate cancer.

**Methods:**

hSef function was studied in *in vitro* and *in vivo* prostate cancer models using stable over-expression clones. Protein expression of hSef was studied in a comprehensive tissue microarray.

**Results:**

Stable over-expression of hSef resulted in reduced *in vitro* cancer cell proliferation, migration and invasive potential. In an *in vivo* xenograft model, the expression of hSef significantly retarded prostate tumour growth as compared with empty vector (*P*=0.03) and non-transfected (*P*=0.0001) controls. Histological examination further showed a less invasive tumour phenotype and reduced numbers of proliferating cells (*P*=0.0002). In signalling studies, hSef inhibited FGF-induced ERK phosphorylation, migration to the nucleus and activation of a reporter gene. Constitutively active Ras, however, was able to reverse these effects, suggesting that hSef exerts an effect either above or at the level of Ras in prostate cancer cells. In a large tissue microarray, we observed a significant loss of hSef protein in high-grade (*P*<0.0001) and metastatic (*P*<0.0001) prostate cancer.

**Conclusions:**

Considered together, the role of hSef in attenuating FGF signalling and evidence of downregulation in advanced tumours argue strongly for a tumour suppressor function in human prostate cancer.

The fibroblast growth factor (FGF) axis is an important promoter of tumourigenesis in many malignancies, including prostate cancer (Grose and Dickson, 2005; Chaffer *et al*, 2007). Work from our own group and others have shown significant over-expression of ligands and receptors in early and late disease (Gnanapragasam *et al*, 2003; Kwabi-Addo *et al*, 2004; Sahadevan *et al*, 2007). However, in addition to these components, a separate layer of endogenous regulators have been shown to significantly modulate the effect of receptor tyrosine kinase (RTK) signalling. These molecules include MAP kinase phosphatases, members of the Sprouty and Spred families and, more recently, similar expression to FGF (Sef). These act as feedback inhibitory checkpoints at key steps in the intracellular signalling cascade (Thisse and Thisse, 2005; Mason *et al*, 2006; Bundschu *et al*, 2007). The role of these regulators in malignancies has attracted much attention with reports of loss of expression in different tumour types (McKie *et al*, 2005; Fong *et al*, 2006; Yoshida *et al*, 2006; Zisman-Rozen *et al*, 2007).

Sef was originally identified by synexpression and association with the FGF axis in embryogenesis (Furthauer *et al*, 2002; Tsang *et al*, 2002). Sef has since been shown to regulate signalling by other growth factors (Xiong *et al*, 2003; Torii *et al*, 2004). The site of Sef action remains controversial. In one of the two original reports in zebrafish, Sef was shown to inhibit ERK activation at the level of MEK (Furthauer *et al*, 2002). In the other report, Sef was shown to immunoprecipitate and colocalise with the FGF receptor (FGFR) (Tsang *et al*, 2002). Work with human and mouse Sef has similarly shown differences with Sef, either acting at the level of Ras interface or downstream at the level of MEK (Kovalenko *et al*, 2003; Xiong *et al*, 2003; Yang *et al*, 2003; Ren *et al*, 2006; Ziv *et al*, 2006). An alternative mechanism has proposed that hSef does not block ERK phosphorylation but rather affects its substrate specificity by spatial regulation into the nucleus or cytoplasm (Torii *et al*, 2004).

We have previously shown that hSef downregulation enhances FGF signalling in prostate cancer cells and evidence of loss of transcript expression in high-grade and metastatic clinical disease in a pilot cohort of patients (Darby *et al*, 2006). This finding has been corroborated by others who have also shown hSef loss in breast, ovarian and cervical cancers (Yang *et al*, 2003; Zisman-Rozen *et al*, 2007). These data raise the possibility that loss of hSef function may be an important common event in the dysregulation of growth factor signalling in epithelial carcinomas. However, the controversies on its mechanism of action suggest that its exact function in a given cell type needs to be carefully characterised. In this study, our aim was to analyse further the functional effect of hSef in a cell model of prostate cancer and test the protein expression pattern of hSef in a large clinical cohort of prostate cancers.

## Materials and methods

### Constructs and stable cell lines

pCDNA3.1-Sef-Myc, pFA2-ELK-1luc and pFR-Luciferase were gifts from Professor Z Chang (Tsinghua University, China) and pHO6T1-H-Ras-HA from Professor J Lunec (Newcastle University, UK). All plasmids were sequence verified before use. DU145 cells were seeded into 90 mm tissue culture dishes and transfected with pCDNA3.1-Sef-Myc or empty vector (Superfect, Qiagen, Crawley, UK) before being placed under G418-sulphate selection for 14–20 days. Individual colonies were removed by trypsinisation and expanded. Cells were maintained in RPMI 1640 media (Sigma, Poole, UK) containing 10% foetal calf serum (FCS), termed full medium (FM). Media lacking FCS was termed basal medium (BM).

### Quantitative real-time PCR (QPCR)

Quantitative real-time PCR was performed using a 7900-HT sequence detection system (Applied Biosystems, Warrington, UK). Total RNA was extracted using Trizol (Invitrogen, Paisley, UK) and reverse transcribed (Promega, Southampton, UK) according to the manufacturer's instructions. QPCR was performed using SYBR Green mastermix (Invitrogen) and primers designed using primer express 2.0 (Applied Biosystems) as follows: hSef forward 5′-CAGAACTTCGGCTTCCGTTT-3′, hSef reverse 5′-CTG CTCACAGGTCTTTCGCTTG-3′, GAPDH forward 5′-CGACCACTTTGTCAAGCTCA-3′ and GAPDH reverse 5′-GGGTCTTACTCCTTGGAGGC-3′. Thermocycle conditions for all PCR reactions were a denaturation stage of 95 °C per 10 min, followed by 95°C for 15 s and 60 °C for 1 min for 40 cycles; a disassociation stage was performed to ensure single amplicon formation. Levels of expression were evaluated using the absolute quantification method normalising against the housekeeping gene GAPDH (ABI technical bulletin 2, www.appliedbiosystems.com).

### Immunofluorescence

Cells were plated at 2000 per well in eight-well BD Falcon slide chambers (Cowley, Oxford, UK). Cells were starved and treated with FGF-1 (100 ng ml^−1^), FGF-2 (10 ng ml^−1^), FGF-8 (100 ng ml^−1^) or FM. Cells were fixed in methanol and primary antibody to p-ERK (Santa Cruz Biotechnology, Santa Cruz, CA, USA) applied in a dilution of 1:50 followed by TRITC-conjugated secondary antibody (Dako, Ely, UK) (1:200). Slides were mounted using DAPI containing mountant (Dako) and visualised using a DMR fluorescent microscope at × 400 magnification (Leica Microsystems, Milton Keynes, UK). In Ras studies, 2 *μ*g of pHO6T1-H-Ras was transfected per well and the above experiments were repeated.

### Proliferation assay

Cells were seeded at a density of 3000 per well in 96-well plates. Cells were starved in BM for 24 h before stimulation with FM and FGF1, FGF2 or FGF8 (in doses of 0, 1, 10 and 100 ng ml^−1^ as appropriate) for 48 h or at the time intervals indicated. A total of 10% WST-1 reagent (Roche, Lewes, UK) was added to the wells and colorimetric change was measured at 450 nm on a Specta Max 250 plate reader (Molecular Devices, Crawley, UK). To test the effects of Ras, 100 ng of pHO6T1-H-Ras were transfected per well and the above experiments were repeated.

### Invasion and scrape assays

A total of 50 000 cells were plated out into BD Falcon migration chambers in BM. FGF-1, FGF-2, FGF-8 or FM was used in the lower chamber as the chemo-attractant with an intervening matrigel layer. Cells were allowed to migrate across a matrigel layer for 16 h in a humidified tissue culture incubator. Non-migrated cells were removed from the upper chamber and cells fixed in methanol for 20 min at −20 °C and then stained with haematoxylin. Membranes were removed with a sterile scalpel and mounted on to slides with DPX and counted using a bright field microscope at × 20 magnification. A minimum of four different fields of view were used to obtain an average count per section. Wound scrape assays were performed by plated cells at a density of 70 000 cells per well in six-well plates and left to adhere overnight. A sterile tip was used to scratch a wound and cells left for 16 h under FM stimulation before being examined using light microscopy.

### Western blotting

Cells were lysed directly using SDS sample buffer containing 10% *β*- mercaptoethanol. Samples were denatured and separated using SDS–PAGE, followed by transfer to nitrocellulose membrane. Membranes were blocked with 5% milk in Tris-buffered saline (TBS; 50 mM Tris-HCl (pH 7.5), 150 mM NaCl) for 1 h, and then incubated with primary antibodies (1:500) in diluent (0.1% milk in TTBS (1% Tween/TBS)) for 1 h at room temperature. Primary antibody complexes were detected using HRP-conjugated secondary antibodies and protein bands were visualised using enhanced chemiluminescence (Amersham, Piscataway, NJ, USA).

### Luciferase reporter assays

In luciferase assays, cells were transfected with a total of 150 ng of DNA per well (24-well plate), comprising 100 ng Elk1-luc or 100 ng pFR-luciferase, and 50 ng *β*-galactosidase reporter, starved in BM for 24 h and then stimulated with FGF1 (0–100 ng ml^−1^) FGF2 (0–10 ng ml^−1^) or FGF8 (0–100 ng ml^−1^) (R&D Systems, Abingdon, UK). Cells were harvested and assayed for luciferase activity corrected for *β*-galactosidase activity according to the manufacturer's guidelines (Promega, Southampton, UK).

### Prostate xenograft studies

*In vivo* experiments were reviewed and approved by the institutional animal welfare committee and performed according to the UKCCCR guidelines. Male athymic nude mice (CD1 nu nu^−1^, Charles River, Margate, Kent, UK) were maintained and handled in isolators under specific pathogen-free conditions. Tumours (DU145 wild type, DU145-EV and DU145-Sef) were generated by implantation of 50 *μ*l of tumour cell suspension subcutaneously into one flank of CD-1 nude mice (1 × 107 cells per animal, *n*=5 animals in each group for each of the two studies). Two-dimensional caliper measurements were made and tumour volume was calculated using the Equation *a*2 × b/2, in which *a* is the smallest measurement and *b* the largest. At the end of study, tumours were harvested and fixed in formalin before paraffin embedding. Sample sections were stained with haematoxylin and eosin or used for immunohistochemistry. Differences in Ki67 stain between tumours were assessed by calculating the positive nuclei in four separate fields of view ( × 200) to derive a mean value for each slide. Three slides for each tumour were assessed for an average score for each tumour.

### Prostate tissue microarray

The tissue microarray used for immunohistochemistry in this study has been previously developed in our unit and reported (Sahadevan *et al*, 2007). Clinical data for the samples included were collected retrospectively and stored in an anonymous database. Cases were selected for this array to ensure a good spread of disease grade, stage and metastatic status. Age-matched benign tissues from patients with no histological diagnosis of CaP were also included. Normal tissues, including breast, kidney, lung and placenta, were included in the microarray. Standard microtome sectioning method was used to cut 5 *μ*m thickness sections.

### Immunohistochemistry and *in situ* hybridisation

Antigen retrieval was achieved using 0.01 M sodium citrate buffer (pH 6.0) and microwaving for 2 min on full power (1000 W) in a pressure cooker. Anti-hSef (R&D systems) and anti-Ki67 (Santa Cruz) monoclonal antibodies were applied in a dilution of 1:500. The Anti-hSef antibody was raised against recombinant human Sef (aa 27–299; Acc AAM77571). Analysis of the sequence confirmed that this antibody would detect the known long and short isoforms of Sef. To confirm antibody specificity, sections incubated with the competing Sef antigen (R&D systems) were used internal negative controls. Secondary labelling was achieved using biotinylated antibodies (Dako). All sections were counterstained with DAB and haematoxylin. Sample sections were viewed by two independent observers and inter-observer agreement was obtained regarding a grading system. The level of Sef expression was assumed to correlate with the strength of the immunoreactivity signal and scored as either absent or weak (−/+), moderate (++) or strong (+++). When two or more signal intensities were present in one case, the predominant signal was taken as the score. In *in situ* hybridisation the cDNA template for the riboprobe was designed from the unique segment of the long isoform (AF494208) and incorporated a T7 and T3 promoter (Yorkshire Biosciences, York, UK). Anti-sense and sense riboprobes were generated after sequence verification. Signals were scored as described above. Signals were scored as described above. The technique of *in situ* hybridisation has been previously described (Gnanapragasam *et al*, 2001).

### Statistical tests

Statistical analysis for *in vitro* and *in vivo* studies was performed using two-tailed Student's *t*-test. Analysis for the clinical data was further performed by correlating hSef expression with clinical parameters using the Kruskal–Wallis test. A *P*-value of ⩽0.05 was taken as significant.

## Results

### hSef inhibits *in vitro* prostate cancer cell proliferation, migration and invasion

**Figure 1:**
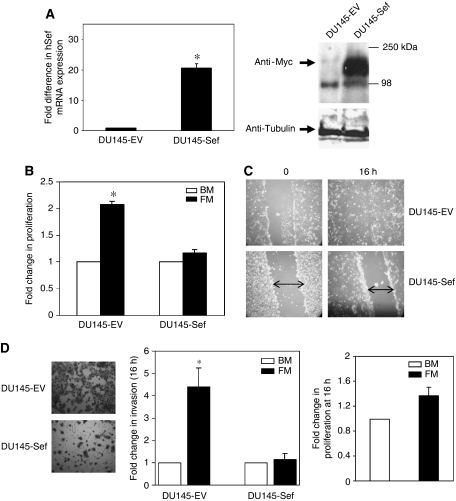
Characterisation and *in vitro* behaviour of hSef expression clones. (**A**) Expression of hSef in the stable clone was assessed at the transcript level using QPCR (left panel) and at the protein level using western blot against the Myc tag (right panel). In transcript assay the fold increase in expression is shown and represents the mean of three studies each performed in triplicate. (**B**) Proliferation assays using serum-enriched media as a stimulus. In these studies the mean of three experiments, each repeated in triplicate, is shown and expressed as a fold increase over un-induced cells. (**C**) In wound scrape assays the extent of cell migration across a cleared line was compared between DU145-EV and DU145-Sef clones at 16 h. (**D**) Invasion assays using serum-enriched media as a stimulus for 16 h. Parallel proliferation experiments were also conducted at 16 h in control DU145 cells to confirm that any change was not due to an increase in cell numbers (right panel). In wound and invasion studies one representative image of three experiments is shown. In invasion assays the results are shown as the mean fold change of these in comparison with DU145-EV cells. Proliferation experiments were conducted as described above (^*^P<0.005 with error bars representing s.d. from the mean). Abbreviations: BM=basal media; FM=full media.

We have previously shown almost undetectable levels of hSef in the DU145 prostate cancer cell line, which is also very sensitive to FGF stimulation (Mehta *et al*, 2001; Darby *et al*, 2006). DU145 cells were stably transfected with empty vector (DU145-EV) or full-length hSef (DU145-Sef). hSef over-expression was confirmed at the transcript level using QPCR and at the protein level using western blot against the Myc tag (Figure 1A). We next analysed the biological effect of Sef transfection. Stimulation with serum-enriched media resulted in a significant increase in DU145-EV cell proliferation (*P*<0.005; Figure 1B). In contrast, DU145-Sef cells failed to respond to stimulation. In wound scrape assays, over-expression of hSef significantly inhibited cell migration across a cleared line (Figure 1C). In invasion assays we again observed similar results with hSef significantly reducing cell movement across a matrigel layer at 16 h (*P*<0.005; Figure 1D, left and middle panel). Parallel proliferation experiments confirmed that the four-fold increase in invasion in control cells was unlikely to be due to increased cell numbers as there was only a modest change in proliferation at 16 h (Figure 1D, right panel). These results suggest that increased hSef expression significantly inhibits prostate cancer cell behaviour *in vitro*.

### Expression of hSef results in a slow-growing and less-invasive *in vivo* tumour phenotype

**Figure 2:**
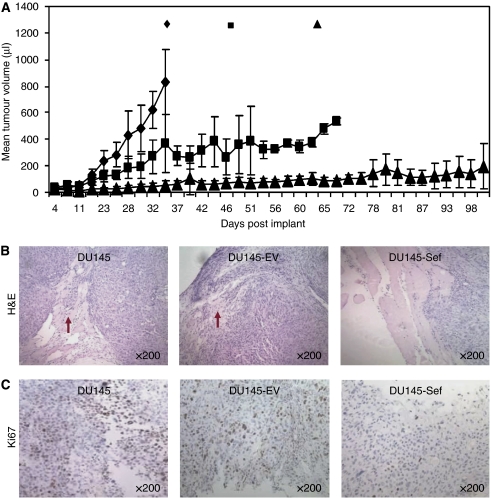
*In vivo* tumour growth and phenotype of hSef over-expressing cells. (**A**) Graph of mean tumour volume changes over time for DU145 wild-type (DU wt), DU145-EV and DU145-Sef clones. One representative of two repeat studies is shown with each point representing the mean volumes of all tumours in the individual group (^*^P=0.0001 and ^**^P=0.03 for mean tumour volume at the end of study comparing DU145-Sef with wild-type DU145 and DU145-EV, respectively). Error bars represent s.d. from the mean. (**B**) H&E stain of representative tumour sections. The red arrows indicate areas of tumour invasion into underlying muscle. (**C**) Immunoassaying for Ki67 as a marker of tumour proliferation. Numbers of positive nuclei were assessed as described in the Materials and Methods and compared between tumours.

To analyse the *in vivo* relevance of hSef expression, parental DU145, DU145-EV and DU145-Sef cells were implanted subcutaneously in nude mice. In these studies we observed a marked difference in tumour growth and behaviour. Wild-type DU145 grew rapidly with a mean final tumour volume of 832 ml by day 34 (Figure 2A). At this time point there was a significant difference in the mean tumour volumes between wild-type DU145 tumours and empty vector controls (326 ml) and DU145-Sef tumours (55 ml; *P*=0.003 and *P*<0.0001, respectively). DU145-EV tumours grew more slowly but progressively enlarged, with all animals killed by day 70 (mean final tumour volume 535 ml). Tumours expressing hSef, however, developed very slowly with mean final tumour volumes (196 ml) significantly lower than the comparative wild-type (*P*=0.0001) and empty vector control (*P*=0.03). No animal in this group required killing before the maximum time allowed for the study (100 days). Microscopy of the harvested tumours revealed extensive muscle invasion by tumours produced by DU145 and DU145-EV cells in keeping with the expected malignant phenotype (Figure 2B). In contrast, DU145-Sef tumours showed a much less aggressive phenotype with minimal invasion of the underlying muscle. Sections of tumour were then immunoassayed for Ki67 as a measure of tumour proliferation. In this study we found numerous proliferating cells in both wild-type and DU145-EV cells (Figure 2C). However, DU145-Sef tumours showed significantly fewer Ki67-positive cells consistent with a slowly proliferating phenotype (*P*=0.0004 compared with wild-type and *P*=0.0002 with DU145-EV). These findings suggest that hSef has a significant inhibitory effect on *in vivo* prostate tumour growth.

### hSef attenuates multiple FGF ligand-induced MAPK activation

**Figure 3:**
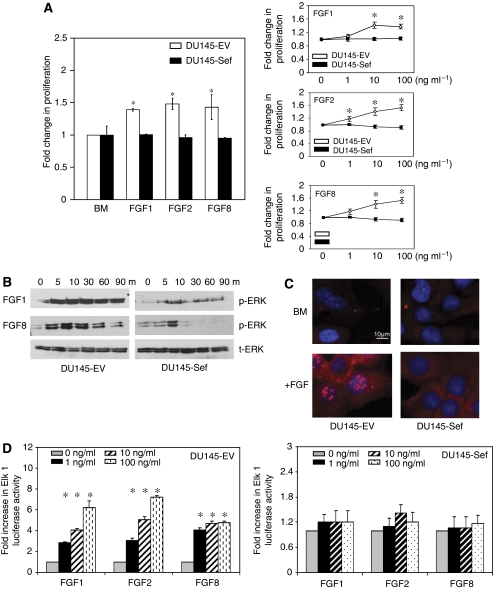
hSef regulation of FGF-induced MAPK signalling. (**A**) Proliferation assays using FGF1, FGF2 and FGF8 in control and hSef-expressing clones (left panel) and at different doses (right panels). The mean of three experiments, each repeated in triplicate, is shown and expressed as a fold change over un-induced cells. (**B**) Phosphorylation of ERK in response to FGF stimulation over timed intervals with corresponding total ERK levels. One representative of three experiments is shown. (**C**) Immunofluorescence for activated ERK with and without 10 ng ml^−1^ FGF2 stimulation. Nuclear expression of ERK is shown by white arrows. One representative of three experiments is shown. (**D**) Co-transfection of an Elk1 reporter was used to test the effect of hSef on gene transactivation. In these studies the mean of three experiments, each repeated in triplicate, is shown and expressed as a fold change in luciferase activity over un-induced cells (^*^P<0.005 with error bars representing s.d. from the mean).

We next tested the effect of hSef expression on FGF-induced signalling in prostate cancer cells. In these studies, hSef blocked cell proliferation in response to different FGFs (Figure 3A, left panel). This inhibitory effect was evident despite increasing levels of the ligand (Figure 3A, right panel). We then tested the effect of hSef on activation of the MAPK pathway. In DU145-EV cells the addition of FGF1 and 8 resulted in rapid phosphorylation of ERK (Figure 3B). However, this response was significantly reduced in cells expressing hSef. In these cells, both the intensity and duration of ERK phosphorylation was reduced. FGF stimulation also resulted in rapid nuclear accumulation of pERK in DU145-EV cells. In contrast, we could not detect pERK in the nuclei of DU145-Sef cells, either with or without ligand stimulation (Figure 3C). An Elk 1 reporter gene was next transfected into our cell models to test whether the observed effect resulted in a change in target gene transcription. In these studies, FGF 1, 2 and 8 enhanced Elk 1 activation in control DU145-EV cells in a dose-dependent manner. However, hSef expression efficiently blocked Elk 1 activation in response to FGFs irrespective of the ligand dose used, confirming the inhibitory effects on ERK activation (Figure 3D).

### The inhibitory function of hSef is reversed by ectopic expression of active Ras

**Figure 4:**
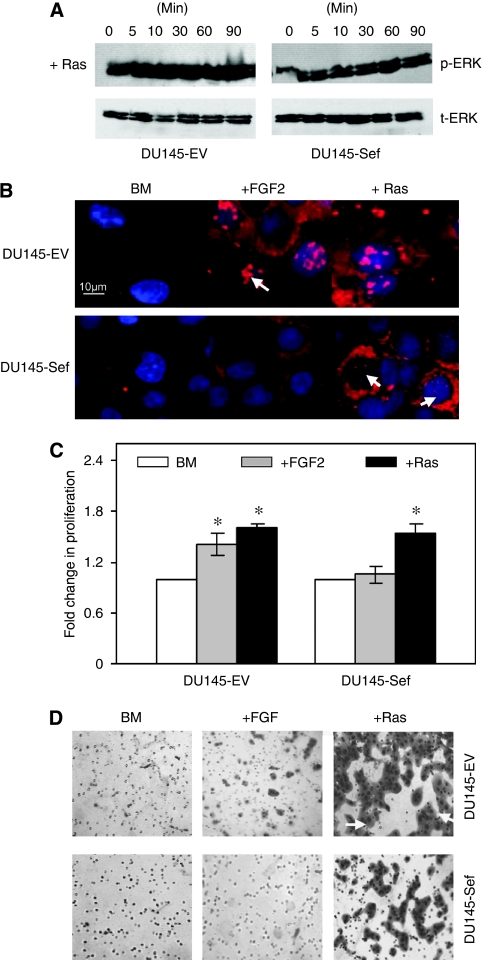
Effect of Ras expression on hSef function. (**A**) Functional effects of Ras expression was confirmed by assaying ERK activation using western blot in DU145-EV and DU145-Sef cells. (**B**) Immunofluorescence for activated ERK in the presence of FGF2 or Ras over-expression. In all these studies one representative of three experiments is shown. (**C**) Proliferation and (**D**) invasion assays, respectively, using BM, BM+FGF2 (10 ng ml^−1^) or BM+Ras transfection. In proliferation studies the mean of three experiments, each repeated in triplicate, is shown and expressed as a fold increase over un-induced cells. In invasion studies one representative of three experiments is shown. One representative of three experiments is shown (^*^P<0.005).

To analyse the site of hSef action in prostate cancer cells, we co-transfected a constitutively active Ras construct. Functional effects were confirmed by the presence of ligand-independent sustained ERK phosphorylation in control DU145-EV cells (Figure 4A). This effect was also observed in DU145-Sef cells, suggesting that active Ras was able to overcome its inhibitory effects on ERK phosphorylation. These findings were further confirmed in IF studies in which the presence of active Ras restored pERK accumulation in the nucleus of DU145-Sef cells (Figure 4B). We next tested whether this translated into a change in cell behaviour. The presence of active Ras reversed the inhibitory effects of hSef and increased cell proliferation to similar levels observed in DU145-EV cells (Figure 4C). A similar effect was observed in invasion assays in which the presence of active Ras significantly enhanced cell movement, whereas FGF stimulation alone failed to have any effect on DU145-Sef cells (Figure 4D). These results suggest that the point of hSef action in prostate cancer cells is likely to be either above or at the level of Ras. Active Ras can overcome the inhibitory effects of hSef in prostate cancer cells.

### hSef protein is downregulated in high-grade and metastatic clinical disease

**Figure 5:**
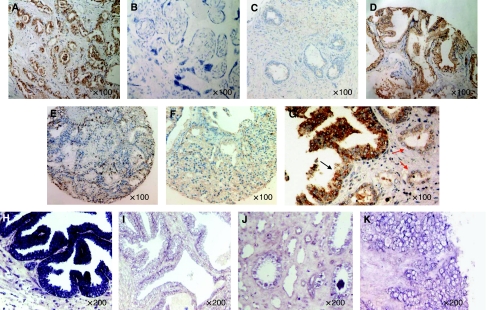
Expression of hSef protein transcript in clinical tissue. (**A**) Strong hSef protein expression in positive control kidney tissue. (**B**) Lack of protein expression in negative control placental tissue. (**C**) Addition of the blocking hSef peptide resulted in a loss of signals confirming antibody specificity. (**D**) Strong positive protein expression in benign prostate glandular epithelium. (**E**) and (**F**) Significantly reduced levels of hSef protein expression in malignant prostate glands. (**G**) Representative section showing positive hSef protein in benign glands (black arrow) and loss of expression in adjacent malignant glands (red arrow). (**H**) *In situ* hybridisation with hSef full-length specific mRNA anti-sense probe showing strong expression in benign prostate glands. (**I**) Lack of signals from hybridisation with a sense probe. (**J, K**). *In situ* hybridisation with hSef full-length mRNA anti-sense probe showing lack of expression in malignant prostate glands.

We next analysed the pattern of hSef protein expression in clinical tissue. The hSef antibody used for the study was first tested in a panel of control tissue. In this analysis, the antibody detected hSef in previously validated positive control kidney sections but not in negative control placenta or in the presence of the blocking peptide (Figures 5A–C, respectively) (Yang *et al*, 2003; Darby *et al*, 2006). In benign biopsies (*n*=43), 74% of cases (32 out of 43) expressed strong levels of hSef and 9% had weak or absent signals (Figure 5D, and Table 1). In contrast, only 21% (37 out of 176) of cancer biopsies (*n*=176) retained strong levels of hSef, whereas 25% had weak or absent protein expression (*P*<0.0001) (Figures 5E and F and Table 1). Areas of absent hSef expression in malignant cells were also frequently found adjacent to strongly positive staining benign glands (Figure 5G). Cancer biopsies were further analysed by Gleason grade and stage; 44% (23 out of 52) of grade 3 cancers retained strong expression of hSef, whereas this figure fell to only 6% (3 out of 53) among grade 5 cancers. Concomitantly, a greater number of high-grade cancers had weak or absent hSef expression (39% (19 out of 49) of grade 5 cancers compared with 6% (3 out of 52) of grade 3 cancers; Table 1A). Similar to our previous transcript findings, there was no association with clinical stage in this study (*P*=0.053). In total, 141 cancers were informative for metastatic status at presentation. hSef protein was weak or absent in 46% (17 out of 37) of biopsies from men with bone metastasis. In contrast, weak or absent signals were only observed in 17% (18 out of 104) of men without metastasis (Table 1A; *P*<0.0001). hSef is known to have a long (full length) and short isoform, and there are currently no isoform-specific hSef antibodies available. The two isoforms are identical except that the shorter isoform lacks a 144 aa sequence in the N-terminal region. To confirm downregulation of the full-length Sef in our clinical series, mRNA *in situ* hybridisation was performed using a probe to a unique sequence of the full-length isoform. In a subset of prostate biopsies (39 cancer and 10 benign samples), we observed strong expression of hSef in benign prostates with anti-sense probes but not with sense probes (Figures 5H and I, respectively). However, the signals were significantly reduced in cancer samples (Figures 5J and K and Table 1B). These results suggest that hSef is closely linked with tumour aggressiveness, with reduced expression being a feature of high-grade and metastatic clinical disease. Protein detection showed loss of total hSef in high-grade cancer biopsies, and *in situ* mRNA studies confirmed parallel loss of the full-length transcript in prostate tumours.

## Discussion

Sef is closely expressed with FGFs in embryogenesis and exerts an effect as a negative feedback regulator in preventing excessive ligand-induced growth (Furthauer *et al*, 2002; Tsang *et al*, 2002). This function is retained in adult tissue, suggesting a continued role in determining the overall effect of cell stimulation by FGFs. The reduced endogenous hSef expression in DU145 cells provides an optimal model in which to analyse its function in prostate cancer. In this cell model, we found that full-length hSef significantly inhibited tumourigenic behaviour *in vitro*. These findings are consistent with our converse observation in a different cell line (PC3) that hSef silencing enhanced cancer cell proliferation and invasion (Darby *et al*, 2006). Thus, these complimentary observations in two separate cell types provide strong evidence of a tumour inhibitory function for hSef in prostate cancer. *In vivo* studies provided further support for this notion as xenograft tumours established from hSef-expressing cell showed very slow tumour growth and a reduced ability to invade adjacent tissue when compared with wild-type or vector only controls.

The site of hSef action is controversial with the key delineation between function at the level of the receptor/Ras or at the level of MEK. In this study the introduction of active Ras reversed the inhibitory effects of hSef and resulted in increased ERK activation and prostate cancer cell proliferation and invasive potential. These results suggest that in prostate cancer cells hSef exerts an effect at the level of the FGFR or at Ras. Functionality at this level is further supported by the consistent finding of Sef interaction (colocalisation and immunoprecipitation) with the FGFR across different species and cell types and its ability to inhibit both FGFR as well as FRS phosphorylation (Tsang *et al*, 2002; Kovalenko *et al*, 2003, 2006; Xiong *et al*, 2003; Yang *et al*, 2003; Ren *et al*, 2007). The ability of hSef to act upstream or at Ras would imply that it should also inhibit alternate pathways activated by FGFR/Ras interaction, which include PI3 kinase (Gioeli, 2005). In keeping with this, both human and mouse Sef have been shown to block FGF induction of the PI3 kinase pathway (Kovalenko *et al*, 2003; Ziv *et al*, 2006). The exact mechanism of how hSef might inhibit FGFR/Ras activation of MAPK signalling in prostate cancer cells is currently unclear. Kovalenko *et al* (2006) have proposed that mSef may act by interfering with FGFR dimerisation and hence prevent receptor phosphorylation and downstream signalling. An alternative mechanism is that hSef interacts directly with and inhibits Ras activation by FGFR at the plasma membrane (Ren *et al*, 2006). Functionality of hSef at this level would also provide an explanation as to the lack of inhibitory function of the short isoform in that the truncated sequence does not localise to the cell membrane. Current work in our group is analysing the exact mechanisms by which Sef might inhibit FGFR/Ras and MAPK signalling in prostate cancer cells.

Only one human Sef (*hSef*) gene has been identified, although two isoforms are known to exist (Rong *et al*, 2006; Ziv *et al*, 2006). Full-length Sef is predicted to contain a signal peptide and transmembrane domain and is ubiquitously expressed in human tissues. The short isoform is thought to be primarily cytosolic and has a more restricted pattern of expression (Preger *et al*, 2004). Both human isoforms have been shown to inhibit RTK signalling in cell line models (Rong *et al*, 2006; Ziv *et al*, 2006). Differences in cell-specific isoform function have been previously reported. In an elegant study Ziv *et al* (2006) showed that full-length hSef inhibited MAPK activation in epithelial cells but not in fibroblast cells. In contrast, the short isoform of hSef inhibited MAPK activation in both cell types. Work with the full-length mouse homologue, however, showed that Sef was capable of blocking FGF-induced cell proliferation in fibroblast cells and that this was associated with decreased phosphorylation of Raf, MEK and ERK (Kovalenko *et al*, 2006). In this study we were only able to focus on the function of full-length Sef and therefore cannot currently comment on the role of the short isoform in prostate cancer cells. An analysis of differences in function between these isoforms would be of great interest and we are developing cell models to undertake this.

hSef transcript expression has been shown to be downreguated in different epithelial malignancies. However, changes at the transcript level may not necessarily translate into a loss of expression at the protein level. In a large clinical cohort, we observed reduced total hSef protein levels in a significant number of cancers compared with benign biopsies. Further analysis of the cancer group showed that downregulation was most evident in high-grade and metastatic tumours. Reduced hSef levels, therefore, do not seem to be an early event in prostate carcinogenesis but is likely to be acquired in the transition to aggressive clinical disease. These findings validate previous reports of reduced hSef transcript expression in aggressive disease (Darby *et al*, 2006). The antibody available to us could not differentiate between the two isoforms of hSef. To corroborate our functional data, we wanted to specifically test whether the full-length isoform of hSef was downregulated in cancer. We therefore studied specific expression of full-length hSef by *in situ* hybridisation using probes specific for the unique segment of the long isoform. In these studies, we observed an identical trend of reduced expression in malignant glands but intact expression in benign glands.

In this study we have primarily analysed the role of hSef in FGF-induced MAPK signalling. However, we would hypothesise that hSef action at the level of the plasma membrane would allow it to interact with different receptors and diverse downstream signals that originate from this level. The profound anti-tumourigenic effect of hSef on xenograft tumours in this study, despite the complex *in vivo* environment, would support this notion. hSef has been reported to interact with both nerve growth factor (NGF) and epidermal growth factor (EGF) signalling (Xiong *et al*, 2003; Torii *et al*, 2004; Ziv *et al*, 2006). Silencing of hSef has further been shown to accelerate EGF-stimulated proliferation of cervical carcinoma cells *in vitro* (Zisman-Rozen *et al*, 2007). More recently, Ren *et al* (2008) reported evidence that hSef interacts and co-localises with the EGF receptor. Intriguingly, in this paper, hSef attenuated EGFR degradation and enhanced EGF-mediated MAPK signalling, adding further diversity in cell-specific function to its role.

The *in vitro*, *in vivo* and clinical results in this study, as well as that of our previous work, strongly support the notion of a tumour suppressor function for hSef. On the basis of these data, we suggest the following model for hSef function: in benign prostate cells, hSef functions as an inducible feedback inhibitor as part of the normal homeostatic mechanism. In these cells, hSef attenuates the effect and duration of receptor signal transduction. It thus limits the effect of mitogenic stimulation by FGFs. In prostate cancer, however, expression of hSef is reduced by mechanisms that are as yet unknown but are common to both isoforms. In these cells, the loss of hSef results in growth factor signalling that is unopposed by this normal regulatory mechanism and can result in un-attenuated downstream signalling. Prostate cancer tumours also frequently over-express FGF ligands and their cognate receptors and this can further enhance mitogenic signalling. The loss of hSef, therefore, is not in itself an oncogenic event but has an important permissive role in enhancing growth factor stimulation in prostate cancer. This hypothesis is supported by others who have similarly found that the loss of hSef can augment receptor tyrosine kinase signalling in cancer cells (Zisman-Rozen *et al*, 2007).

Multiple alterations are known to occur in the FGF axis in prostate cancer. The contribution that loss of Sef might make in the context of concomitant changes in FGF ligands and receptors is therefore of great interest and is currently being analysed in our unit (manuscript in preparation). The current tissue microarray has previously been used to profile FGFR1 and FGFR4 expression (Sahadevan *et al*, 2007). In a preliminary analysis of our series, we tested whether loss of Sef may be related to FGFR1 or FGFR4 over-expression. Sef loss was observed in 57% of tumours with weak or moderate levels of FGFR1 and also in 38% of tumours with strong FGFR1 expression (*P*=0.27). Similarly, loss of Sef was observed in 44% of tumours with weak or moderate FGFR4 expression and also in 41% of tumours with strong FGFR4 expression (*P*=0.81). These data suggest that Sef downregulation does not seem to be associated with alterations in FGFR expression in prostate cancer. We next tested whether concomitant loss of Sef and increased FGFR expression may have relevance to tumour behaviour. In tumours with strong FGFR1 expression but weak or absent Sef levels, 71% had evidence of bone metastasis. In contrast, in tumours with strong FGFR1 expression as well as strong Sef expression, only 24% had evidence of bone metastasis (*P*<0.001). A similar observation was made when tumours with strong FGFR4 expression was stratified by differences in Sef expression level. These results support the notion that the loss of Sef may have a key role in enhancing FGF signalling and promoting an aggressive clinical phenotype.

The mechanism that downregulates hSef in cancer cells is currently unknown. A potential mechanism may be by DNA methylation, as has been shown with Sprouty 2 (McKie *et al*, 2005). Another possibility is alterations at the genomic level. In this context, Braga *et al* (2002) have previously reported frequent LOH in chromosome 3p in the locality in which the hSef gene is found. Targeting the mechanisms that downregulate hSef may therefore be a useful method to restore expression levels and its inhibitory effect on FGF stimulation. Assessing tumour expression of hSef, however, may provide an idea of the likely effectiveness of targeted receptor inhibition and help improve growth factor-based therapy for clinical prostate cancer. Our ongoing work is focused on addressing these issues as well as on analysing the role of the short isoform and the exact mechanism by which hSef has its function.

## Figures and Tables

**Table 1:** Analysis of hSef expression in association with clinical disease parameters

	**hSef protein**			
**All tissue samples (*n*=219)**	**−/+**	**++**	**+++**	
*(A)*				
Benign (*n*=43)	4	7	32	
Cancer (*n*=176)	45	94	37	*P*<0.0001
				
*Cancer tissues samples (*n=*176)*				
Gleason grade 3	3	26	23	
Gleason grade 4	23	41	11	*P*<0.0001
Gleason grade 5	19	27	3	
Stage 1	11	32	16	
Stage 2	6	18	5	
Stage 3	15	17	5	
Stage 4	7	13	2	*P*=0.053
Bone metastasis	17	19	1	
No metastasis	18	56	30	*P*<0.0001
				
*(B)*				
*hSef mRNA (full length)*				
Benign (*n*=10)	2	3	5	
Cancer (*n*=10)	19	13	7	*P*=0.04
				
Gleason grade 3	2	3	4	
Gleason grade 4	4	5	3	
Gleason grade 5	13	5	0	*P*=0.006

Abbreviation: Stage=local tumour stage.

A: Protein expression of total hSef analysed by clinical parameters in 219 tissue samples.

B: Specific hSef full-length isoform expression by *in situ* hybridisation in a subset (*n*=49) of benign and cancer tissue samples.
